# Different types of bullae of limbs with necrotizing fasciitis predict different outcome: a prospective study

**DOI:** 10.1007/s15010-020-01559-5

**Published:** 2021-01-03

**Authors:** Tsung-Yu Huang, Yao-Hung Tsai, Liang-Tseng Kuo, Wei-Hsiu Hsu, Cheng-Ting Hsiao, Chien-Hui Hung, Wan-Yu Huang, Han-Ru Wu, Hui-Ju Chuang, Yen-Yao Li, Kuo-Ti Peng

**Affiliations:** 1 Division of Infectious Diseases, Department of Internal Medicine, Chang Gung Memorial Hospital, Chiayi, Taiwan; 2 Graduate Institute of Clinical Medical Sciences, College of Medicine, Chang Gung University, Taoyuan, Taiwan; 3 Department of Nursing, Chang Gung University of Science and Technology, Chiayi Campus, Chiayi, Taiwan; 4 Department of Orthopedic Surgery, Chang Gung Memorial Hospital, No. 6, West section, Chia-Pu Road, Pu-Zih city, Chia-Yi County, Taiwan 61363; 5 Department of Chinese Medicine, School of Medicine, Chang Gung University, Chiayi, Taiwan; 6 College of Medicine, Chang Gung University, Taoyuan, Taiwan; 7 Department of Emergency Medicine, Chang Gung Memorial Hospital, Chiayi, Taiwan; 8 Division of General Surgery, Department of Surgery, Chang Gung Memorial Hospital, Chiayi, Taiwan; 9 Department of Pharmacy, Chang Gung Memorial Hospital, Chiayi, Taiwan; 10 Department of Laboratory Medicine, Chang Gung Memorial Hospital, Chiayi, Taiwan

**Keywords:** Necrotizing fasciitis, Hemorrhagic bullae, Vibrio infection, Bacteremia, Skin necrosis

## Abstract

**Study objective:**

Necrotizing fasciitis (NF) is an uncommon life-threatening necrotizing skin and soft tissue infection. Bullae are special skin manifestations of NF. This study was conducted to analyze the differences between different types of bullae of limbs with NF for providing the information to emergency treatment.

**Methods:**

From April 2015 to August 2018, patients were initially enrolled based on surgical confirmation of limbs with NF. According to the presence of different bullae types, patients were divided into no bullae group (Group N), serous-filled bullae group (Group S), and hemorrhagic bullae group (Group H). Data such as demographics, clinical outcomes, microbiological results, presenting symptoms/signs, and laboratory findings were compared among these groups.

**Results:**

In total, 187 patients were collected, with 111 (59.4%) patients in Group N, 35 (18.7%) in Group S, and 41 (21.9%) in Group H. Group H had the highest incidence of amputation, required intensive care unit care, and most patients infected with *Vibrio* species. In Group N, more patients were infected with *Staphylococcus* spp. than Group H. In Group S, more patients were infected with β-hemolytic *Streptococcus* than Group H. Patients with bacteremia, shock, skin necrosis, anemia, and longer prothrombin time constituted higher proportions in Group H and S than in Group N.

**Conclusions:**

In southern Taiwan, patients with NF accompanied by hemorrhagic bullae appear to have more bacteremia, *Vibrio* infection, septic shock, and risk for amputation. If the physicians at the emergency department can detect for the early signs of NF as soon as possible, and more patient’s life and limbs may be saved.

## Introduction

### Background

Necrotizing fasciitis (NF) is an extremely rare and fulminant necrotizing skin and soft tissue infection (NSSTI) characterized by rapidly progressive necrosis in the subcutaneous tissues, especially the superficial and deep fascia [1–5]. The clinical features of this infection include hemorrhagic bullae, subcutaneous bleeding, purpura, frank skin necrosis, and gangrene [6–9]. In general, hemorrhagic bullae are extremely rare and considered as an important skin manifestation of *Vibrio* infection [10–17]. In addition to infectious diseases, other severe diseases may also manifest the same characteristics, such as vascular disorders, autoimmune diseases, drug or hypersensitivity reactions [18–21].

In southern Taiwan, based on the location near the tropical zone and ocean, there have higher incidence of *Aeromonas* and *Vibrio* bacteremia infections than other areas of Taiwan [22, 23]. Chang Gung Memorial Hospital-Chiayi (CGMH-Chiayi) is a regional hospital which is situated on the western coast of southern Taiwan. For further exploration the *Aeromonas* and *Vibrio* infections and investigation the best therapeutic methods, our team, the “Vibrio NSSTIs Treatment and Research (VTR) Group,” were established at CGMH-Chiayi since 2004, is a professional medical group and specialized in treating and investigating *Vibrio* infectious disease [24, 25]. Our previous studies had reported numerous results, including *Vibrio* NF [11–15, 26–28], *Vibrio* cerebritis [29], *Vibrio* keratitis [30], and *Aeromonas* NF [25] to afford important information for these of infective diseases.

Although the bullae is the importance signs for NF, especially on infectious disease, there is rarely discussed in the literature for the difference in different types of bullae of NF. Therefore, we designed this study to evaluate the demographic data, clinical outcomes, clinical presentations, and laboratory findings of patients with NF when they arrived at the emergency department (ED). The results of this research have benefits for physicians conduct initial diagnosis and consider empirical antimicrobial therapy for emergency patients with NF and bullae.

## Materials and methods

### Setting and study design

**Fig. 1:**
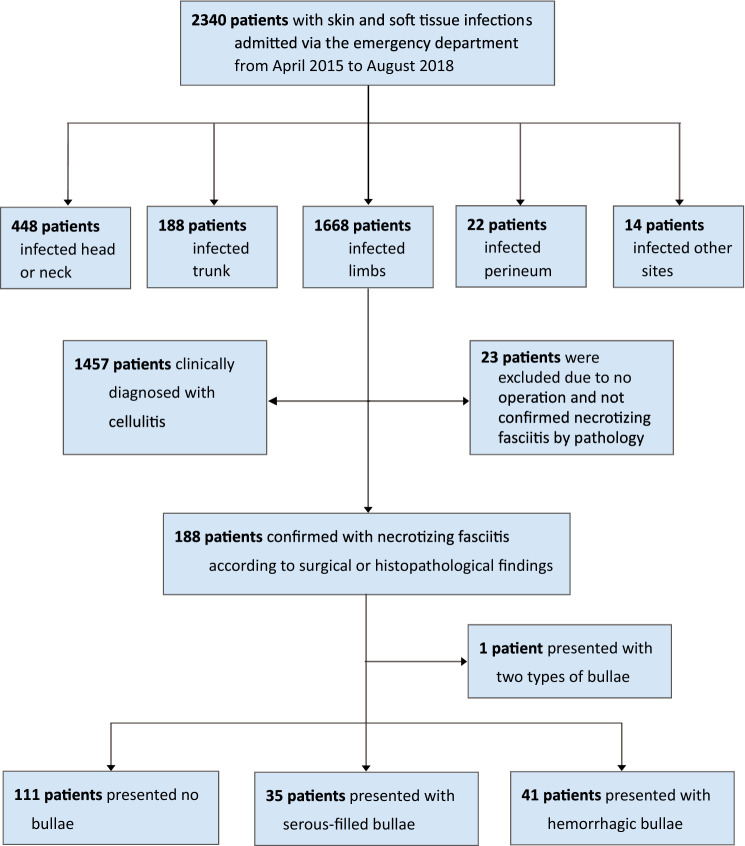
Flowchart of 188 patients with necrotizing fasciitis with different types of bullae

This was a prospective study performed by the VTR Group at CGMH-Chiayi from April 2015 to August 2018. During this period, those patients who were admitted from the ED and diagnosed with skin and soft tissue infections were initially enrolled in this study (Fig. 1).

### Selection of participants

Patients with NF of limbs were enrolled in the study using the following criteria: (1) NF was defined based on surgical findings, including the presence of grayish necrotic skin, subcutaneous fat and fascia, no resistance of normally adherent fascia to digital blunt dissection, and a purulent discharge resembling foul-smelling dishwater [13, 24, 25, 31], (2) Availability of histopathological tissue specimens to confirm the diagnoses [6, 25, 32], and (3) Presence of only infected limb. Bullae can be divided into hemorrhagic or clear [24]. These types of bullae were distinguished according to the patient’s medical records and picture assessment by teamwork. Patients having two types of bullae were excluded from this study.

Monomicrobial infection was diagnosed by isolating single pathogenic bacteria, and polymicrobial infections were diagnosed by isolating more than one pathogenic bacterium from soft tissue lesions and/or blood collected immediately after the patient’s arrival at the ED or during surgery [13, 24, 31].

### Demographic data, clinical presentations, and laboratory findings

Patients with NF of limbs were divided into no bullae group (Group N), serous-filled bullae group (Group S), and hemorrhagic bullae group (Group H) according to the different types of bullae. Data such as demographics, comorbidities, microbiological results, presenting signs and symptoms, laboratory findings, and clinical outcomes were recorded and compared among these three groups.

### Statistical analysis

Continuous data were compared using one-way analysis of variance, and a Tukey post hoc test was performed for multiple comparisons (*p* < 0.05). All statistical calculations were performed using the Statistical Package for the Social Sciences Windows, version 18.0 (SPSS, Chicago, IL, USA) for Windows, and all values were reported as mean ± standard deviation.

## Results

### Study population

Between April 2015 and August 2018, 188 patients admitted via the ED were surgically confirmed to have NF of limbs (Fig. 1). One patient presenting with mixed-type bullae formation were excluded, and the remaining 187 people were divided into the following three groups: 111 (59.4%) patients in Group N, 35 (18.7%) in Group S, and 41 (21.9%) in Group H.

### Demographic data

Group H and Group S were characterized by higher Acute Physiology and Chronic Health Evaluation (APACHE) II scores than Group N. Patients in Group H were characterized by a higher incidence of chronic liver dysfunction, chronic kidney disease, cerebrovascular accidents, and malignant disease than those in Group N (Table 1).

**Table 1:** Demographic data and clinical outcomes of 187 patients with necrotizing fasciitis between the three groups

Variable	Group N^a^ (*n* = 111)	Group S^b^ (*n* = 35)	Group H^c^ (*n* = 41)
Age (years)	64.0 ± 15.5	68.4 ± 10.8	70.3 ± 13.7†
Gender, male	75 (67.6)	22 (62.9)	27 (65.9)
APACHE^d^ II score	11.5 ± 5.1	14.9 ± 5.8†	16.8 ± 5.3†
Involved lower extremities	66 (59.5)	20 (57.1)	33 (80.5)†§
Seawater, seafood contact history or exposure to farm	46 (41.4)	14 (40.0)	15 (36.6)
*Underlying chronic diseases*			
Alcoholism	27 (24.3)	5 (14.3)	8 (19.5)
Chronic liver dysfunction	34 (30.6)	16 (45.7)	20 (48.8)†
HBV infection	18 (16.2)	5 (14.3)	8 (19.5)
HCV infection	22 (19.8)	12 (34.3)	15 (36.6)†
Liver cirrhosis	25 (22.5)	7 (20.0)	9 (22.0)
Chronic kidney disease	26 (23.4)	8 (22.9)	17 (41.5)†
Cardiovascular disease	18 (16.2)	5 (14.3)	10 (24.4)
Cerebrovascular accident	3 (2.7)	2 (5.7)	6 (14.6)†
Diabetes mellitus	46 (41.4)	14 (40.0)	18 (43.9)
Gout	6 (5.4)	5 (14.3)	1 (2.4)
Malignancy	15 (13.5)	5 (14.3)	11 (26.8)†
Peripheral vascular disease	4 (3.6)	0 (0)	2 (4.9)
*Clinical outcomes*			
Mortality	10 (9.0)	5 (14.3)	7 (17.1)
Amputation	6 (5.4)	2 (5.7)	7 (17.1)†
Mortality or amputation	14 (12.6)	7 (20)	13 (31.7)†
Postoperative intubation	11 (9.9)	5 (14.3)	16 (39.0)†
Need ICU^e^ care	33 (29.7)	12 (34.3)	23 (56.1)†§
Number of debridements	2.6 ± 1.3	2.7 ± 1.3	3.2 ± 1.9†
Hospital stay (days)	31.1 ± 17.1	37.9 ± 28.6	36.7 ± 20.7

Data were presented as mean (standard deviation) or frequency (%). * *p*-value < 0.05

Abbreviations: ^a^Group N: no bullae group, ^b^Group S: serous-filled bullae group, ^c^Group H: hemorrhagic bullae group, ^d^*APACHE* Acute Physiology and Chronic Health Evaluation, ^e^*ICU* intensive unit care. Data were presented as mean (standard deviation); Data were compared with ANOVA, †*p* < 0.05 vs. no bullae in the Tukey post hoc test; §*p* < 0.05 vs. serious bullae in the Tukey post hoc test

### Clinical outcomes

Patients in Group H had a higher incidence rate of amputations (17.1% vs. 5.4%) and a higher number of debridements (3.2 vs. 2.6) than those in Group N. Group H patients also had a higher incidence of postoperative intubation (39.0% vs. 9.9%) than patients in Group N and required intensive care unit (ICU) care (56.1% vs. 34.3% vs. 29.7%) than patients in Groups S and N (Table 1).

### Microbiological results

Table 2 shows the microbiological findings of the 187 NF cases. A higher incidence of blood-stream infections was observed in Group H than in Groups S and N (41.5% vs. 25.7% vs. 24.3%). Among the 187 patients, 101 (54.0%) had monomicrobial infection, 37 (19.8%) had polymicrobial infection, and 49 (26.2%) had culture-negative NF. In Group H, more patients were infected with Gram-negative monomicrobial bacterium, especially *Vibrio* species than those in the other two groups. However, the incidence of Gram-positive monomicrobial bacterium was lesser in Group H than in Groups N and S. In Group N, more patients were infected with *Staphylococcus* spp. (19.8% vs. 4.9%). than Group H. And in Group S, more patients were infected with β-hemolytic *Streptococcus* (17.1% vs. 0%) than Group H.

**Table 2:** Microbiological results of 187 necrotizing fasciitis limbs between the three groups

Variable	Group N (*n* = 111)	Group S (*n* = 35)	Group H (*n* = 41)
Blood-stream infection	27 (24.3)	9 (25.7)	17 (41.5)†§
Monomicrobial infection	58 (52.3)	19 (54.3)	24 (58.5)
Gram-negative monomicrobial infection	27 (24.3)	8 (22.9)	22 (53.7)†§
*Vibrio* spp*.*	19 (17.1)	6 (17.1)	15 (36.6)†§
*Vibrio vulnificus*	17 (15.3)	6 (17.1)	14 (34.1)†§
*Vibrio cholerae non-O1*	1 (0.9)	0 (0)	1 (2.4)
*Vibrio parahaemolyticus*	1 (0.9)	0 (0)	0 (0)
*Aeromonas* spp*.*	4 (3.6)	2 (5.7)	4 (9.8)
*Aeromonas hydrophila*	4 (3.6)	2 (5.7)	3 (7.3)
* Aeromonas sobria*	0 (0)	0 (0)	1 (2.4)
*Enterobacter cloacae*	1 (0.9)	0 (0)	1 (2.4)
*Escherichia coli*	1 (0.9)	0 (0)	0 (0)
*Klebsiella pneumoniae*	1 (0.9)	0 (0)	0 (0)
*Pseudomonas aeruginosa*	1 (0.9)	0 (0)	1 (2.4)
*Shewanella putrefaciens*	0 (0)	0 (0)	1 (2.4)
Gram-positive monomicrobial infection	31 (27.9)	11 (31.4)	2 (4.9)†§
*Staphylococcus* spp.	22 (19.8)	5 (14.3)	2 (4.9)†
MRSA^a^	12 (10.8)	2 (5.7)	1 (2.4)
MSSA^b^	9 (8.1)	2 (5.7)	1 (2.4)
Coagulase-negative staphylcoccus	1 (0.9)	1 (2.9)	0 (0)
*Streptococcus* spp.	9 (8.1)	6 (17.1)	0 (0)§
*Streptococcus* species	3 (2.7)	0 (0)	0 (0)
* Streptococcus group non-ABD*	2 (1.8)	2 (5.7)	0 (0)
*Streptococcus pyogenes*	1 (0.9)	1 (2.9)	0 (0)
*Streptococcus agalactiae*	1 (0.9)	1 (2.9)	0 (0)
*Streptococcus dysgalactiae* subsp. *equisimilis*	1 (0.9)	2 (5.7)	0 (0)
*Anaerobic bacteria*			
*Peptostreptococcus* sp	1 (0.9)	0 (0)	0 (0)
Polymicrobial infection	22 (19.8)	3 (8.6)	12 (29.3)§
Culture-negative necrotizing fasciitis	31 (27.9)	13 (37.1)	5 (12.2)†§

Abbreviations: ^a^*MRSA* methicillin-resistant *Staphylococcus aureus*, ^b^*MSSA* methicillin-sensitive *Staphylococcus aureus*

Data were presented as frequency (%).; Data were compared with ANOVA, †*p* < 0.05 vs. no bullae in the Tukey post hoc test; §*p* < 0.05 vs. serious bullae in the Tukey post hoc test

### Clinical presentations

Patients in Group H has a shorter duration of symptoms or signs at presentation than those in Group N (Table 3). There were no significant differences in the presentation of a fever, tachycardia (heartbeat > 100/min), tachypnea (respiratory rate > 20/min), swelling, and an erythematous lesion among the three groups. However, Group H had a higher proportion of patients presenting with shock (mean arterial pressure < 65 mmHg, 31.7% vs. 14.3% vs. 10.8%) and skin necrosis (26.8 vs. 2.9% vs. 7.2%) than Groups S and N (Table 3). In Group N, there were more patients with a painful lesion (98.2% vs. 94.3% vs. 85.4%) than those in Groups S and H.

**Table 3:** Clinical presentations of 187 patients with necrotizing fasciitis between the three groups

Variable	Group N (*n* = 111)	Group S (*n* = 35)	Group H (*n* = 41)
The duration of symptoms/signs (days)	2.9 ± 2.9	2.1 ± 1.3	2.0 ± 2.2†
*Systemic symptoms/signs*			
Fever (> 38 °C)	35 (31.5)	6 (17.1)	12 (29.3)
Tachycardia^a^	58 (52.3)	20 (57.1)	18 (43.9)
Tachypnea^b^	27 (24.3)	8 (22.9)	15 (36.6)
Shock^c^	12 (10.8)	5 (14.3)	13 (31.7)†§
*Limbs symptoms/signs*			
Swelling	109 (98.2)	35 (100.0)	40 (97.6)
Pain or tenderness	109 (98.2)	33 (94.3)	35 (85.4)†§
Erythema	102 (91.9)	33 (94.3)	35 (85.4)
Skin necrosis	8 (7.2)	1 (2.9)	11 (26.8)†§

Data were presented as mean (standard deviation) or frequency (%). ^a^Tachycardia: heartbeat > 100/min, ^b^Tachypnea: respiratory rate > 20/min, ^c^Shock: mean arterial pressure < 65 mmHg. Data were presented as mean (standard deviation); Data were compared with ANOVA, †*p* < 0.05 vs. no bullae in the Tukey post hoc test; §*p* < 0.05 vs. serious bullae in the Tukey post hoc test

### Laboratory findings

The band forms of leukocytes of more than 10%, hemoglobin level < 10 g/dL, and thrombocytopenia (< 15 × 10^4^/µL) were found more frequently in Group H than in Group N (Table 4). The serum lactate values in Group H were significantly higher than those in Group N. The creatinine values in Groups S and H were significantly higher than those in Group N. In Group H, the prothrombin time values were higher than those in the other two groups.

**Table 4:** Laboratory findings of 187 patients with necrotizing fasciitis between the three groups

Variable	Group N (*n* = 111)	Group S (*n* = 35)	Group H (*n* = 41)
Total WBC^a^ count			
Leukocytosis (≧ 12,000/µL)	69 (62.2)	24 (68.6)	22 (53.7)
Leukopenia (≦ 4000/µL)	5 (4.5)	1 (2.9)	2 (4.9)
Differential count			
Band forms > 10%	13 (11.7)	8 (22.9)	11 (26.8)†
Neutrophilia (> 7500/µL)	88 (79.3)	27 (77.1)	28 (68.3)
Lymphocytopenia (< 1000/µL)	21 (18.9)	8 (22.9)	11 (26.8)
Hemoglobin (< 10 g/dL)	7 (6.3)	5 (14.3)	13 (31.7)†§
Thrombocytopenia (< 15 × 10^4^/µL)	40 (36.0)	15 (42.9)	23 (56.1)†
C-reactive protein (< 150 mg/L)	61 (55.0)	16 (45.7)	25 (61.0)
Hypoalbuminemia (< 2.5 g/dL)	10 (9.0)	3 (8.6)	7 (17.1)
Lactate (mg/dL)	22.5 ± 20.9	22.4 ± 13.0	32.4 ± 25.1†
Creatinine (μmol/L)	129.0 ± 105.1	208.1 ± 255.8†	215.9 ± 178.7†
Glucose (mmol/L)	10.4 ± 6.7	10.3 ± 5.3	9.9 ± 7.2
Sodium (mmol/L)	134.7 ± 3.3	134.5 ± 2.2	134.8 ± 4.1
PT^b^ (seconds)	11.5 ± 2.6	11.9 ± 2.5	13.8 ± 5.9†§
Total bilirubin (mg/dL)	1.7 ± 3.0	1.5 ± 1.4	2.6 ± 4.4

Data were presented as mean (standard deviation) or frequency (%). Abbreviations: ^a^WBC: white blood cell; ^b^PT: Prothrombin time. Data were presented as mean (standard deviation); Data were compared with ANOVA, †*p* < 0.05 vs. no bullae in the Tukey post hoc test; §*p* < 0.05 vs. serious bullae in the Tukey post hoc test

## Discussion

Hemorrhagic bullae are small vessel involvement in the dermis that can result in necrosis of overlying skin with associated blisters and extravasation of red blood cells [18]. With the evolution of the infective condition of NF, ischemic necrosis of the skin ensues accompanied by gangrene of the subcutaneous fat, dermis, and epidermis, manifesting progressively as bullae formation, ulceration, and skin necrosis [6]. Approximately 13.3–44.9% of NF cases present with bullae lesions [6, 8, 24, 33, 34], and 8.3–32.6% present with hemorrhagic bullae [8, 31, 34, 35] have reported in literature. In the present study, 40.6% of NF cases manifested bullae appearance and 21.9% of them presented with hemorrhagic bullae. In general, serous-filled bullae are considered to occur in the second stage [7], and hemorrhagic bullae occur in the late-stage of NF [7, 11, 29]. However, the sequences of phenomenon stage are not absolute, it appears that patients with NF presenting with hemorrhagic bullae do not necessarily present with serous-filled bullae. However, the assessment of bullae was only performed when the patient arrived at the ED, and most patients with necrotizing fasciitis received surgical intervention immediately. It was difficult to understand the types of subsequent bullae formation. Pathogen isolated from the mixed bullae patient was methicillin-sensitive *Staphylococcus aureus*. We should pay attention to the changes in bullae in further study.

The overall mortality rate of NF has been reported to be 12.1–76% [2, 6, 7, 31, 33–38]. The mortality rates of patients presenting with hemorrhagic bullae were 19% among overall NF cases [14], 38.5% in *Vibrio vulnificus* skin and soft tissue infections [10], and 46.2% in *Aeromonas* NF cases [25]. But no articles were reported about the mortality rate for NF presenting with serous-filled bullae. In the present study, although the patients in the hemorrhagic bullae and serous-filled groups had a more serious clinical illness with a higher APACHE II score than those in the no bullae group (Table 1), no significantly high mortality was found in the hemorrhagic bullae group. Although the overall amputation rate in NF cases has been found to be 4.7–22.5% [6, 7, 31, 33, 35–38], more people with hemorrhagic bullae must suffer the fate of amputation than other tested groups of this study. Approximately 60.9–66.7% of patients with NF require postoperative intubation is limited literature [31, 39]. Moreover, previously studies also indicated that 45–100% of patients with NF require ICU management [8, 31, 36, 39]. In our study, more patients in the hemorrhagic bullae group required postoperative intubation (39.0%) and ICU care (56.1%) which are higher than other types of bullae. Hemorrhagic bullae appeared to be a manifestation of the severe clinical status, required more critical care, and appeared to have a poor prognosis. Serous-filled bullae also represented with patients having higher mortality, amputation rates, and more serious clinical status, including higher rates for postoperative intubation and need ICU care. But these critical conditions for the serious serous-filled group were less than the hemorrhagic bullae group. An average of 2.6–3.3 operative debridement procedures per patient were necessary to control this fulminant infection [7, 33, 35]. In our study, patients presenting with hemorrhagic bullae definitely required aggressive debridement compared with those in the no bullae group. In addition, patients with NF were susceptible to have a combination of several important underlying chronic disorders, especially chronic liver disease, chronic kidney disease, diabetes mellitus, malignant disease, and peripheral vascular disease [14, 31, 33, 37, 39, 40], and have poor outcomes as reported in previous studies [27, 31, 33, 34, 37, 38]. In the current study, we further found that higher proportions of chronic hepatic dysfunction, chronic kidney disease, stroke, and malignant disease in the hemorrhagic bullae group than other groups, especially the differences could be significantly obtained in the patients without bullae. A similar phenomenon was found in our previous research, and this condition may lead to vascular sclerosis [24]. The vascular sclerosis may cause stroke—a vascular ischemic disease and subsequently may tend to cause ischemic necrosis of the skin and present with hemorrhagic bullae.

Bacteremia was found to be associated with increased mortality related to NF [34, 41], especially when infected with Gram-negative pathogens accompanied by septic shock [24, 25, 40]. Patients in the hemorrhagic bullae group tended to have a concurrent blood-stream infection, infection with Gram-negative monomicrobial bacterium, and *Vibrio* species compared with patients in the other two groups. Hemorrhagic bullae generally develop in infections with *Vibrio* species at the time of admission or within 24 h of hospitalization and became more severe every hour [10]. Yet, there was no report about the interval duration between the occurrence of serous-filled bullae and infectious pathogens for NF.

**Fig. 2:**
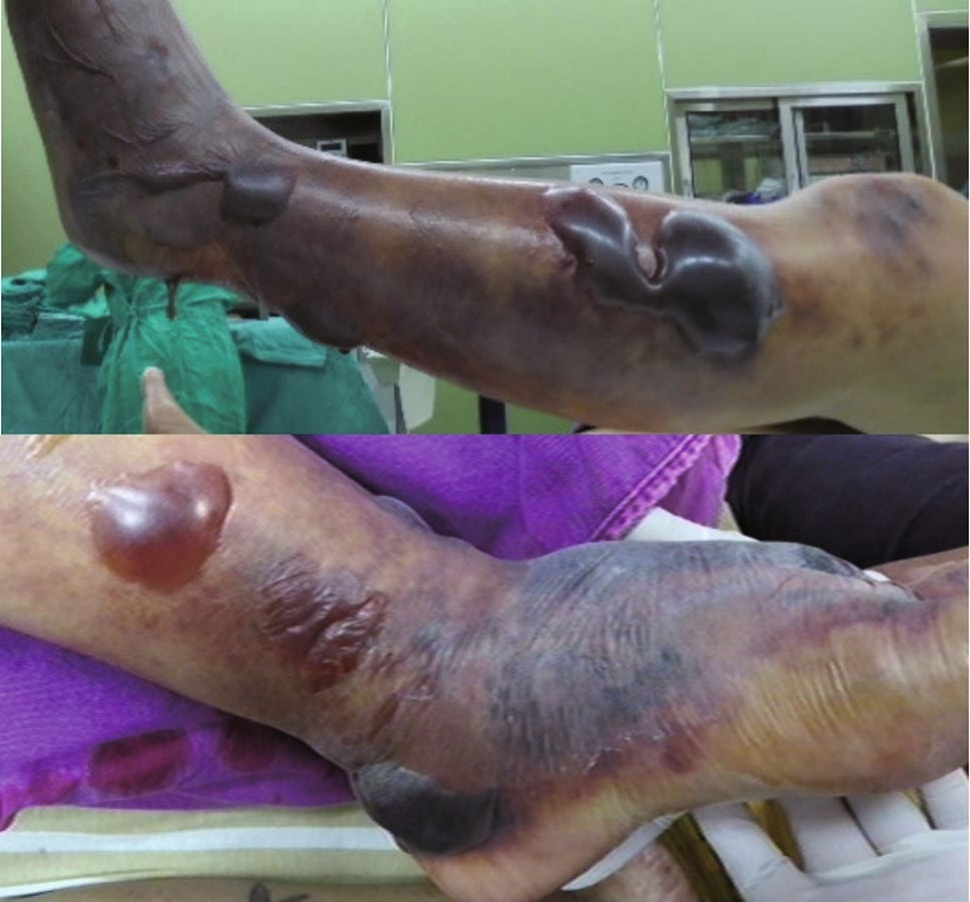
A 46-year-old male with a history of decompensated liver cirrhosis—Child C, alcoholism, hepatitis B, hepatoma, and end-stage renal disease receiving regular hemodialysis had left lower limb pain and conscious disturbance for one day. The left lower leg developed odor smell, hemorrhagic bullae, diffuse purpura, and necrotic skin lesions with diffuse oozing on the bed in the operation room. After fasciotomy and debridement, the culture of blood, tissue, pus, and wound specimens confirmed *Aeromonas hydrophila*; however, this patient died on the 10th day after admission owing to progressive septic shock, esophageal variceal bleeding, and multiple organ failure

There were 87.8% of patients in the hemorrhagic bullae group were infected by microorganisms. An interesting finding was that *Vibrio* could manifest any one type of bullae but appeared to be more prone to have hemorrhagic bullae (Table 2). However, hemorrhagic bullae were not an exclusive feature of *Vibrio* infection, and they could also be found in patients infected with bacteria other than *Vibrio* species, such as *Aeromonas*, β-hemolytic *Streptococcus*, and *Staphylococcus* spp. in NF cases [14, 25, 31]. Approximately 37–65.1% of primary *Vibrio* septicemia cases [10, 42], 41–68.8% of *Vibrio* skin and soft tissue infection cases [10, 13, 42], and 38.2% of *Aeromonas* NF cases [31] can develop hemorrhagic bullae. In the past experiences of our team, patients with hemorrhagic bullae and skin necrosis appearance may have an increased incidence of mortality (Fig. 2).

Although different microbial infections could emerge in different bullae or with no bullae. Based on our limited data, we assumed that if a patient has a high suspicion of having NF in the ED, the hemorrhagic bullae appearing on the skin should be highly suspected of *Vibrio* infection and the serous-filled bullae should imply a *Streptococcal* infection; however, if there were no bullae formation, it may be possible as a *Staphylococcal* infection.

More patients presenting with hemorrhagic bullae appeared to initially present with septic shock and skin necrosis than the other two groups. There were 12.1–64.3% of patients with NF who initially presented with septic shock [6, 27, 31, 33–35, 38, 40]. Patients with hypotension were significantly associated with mortality [35, 38], especially when infected with *Vibrio* or *Aeromonas* species [11, 13, 25, 26]. In the present study, more patients presented with shock in the hemorrhagic bullae group, and this result may be due to more Gram-negative monomicrobial bacterium and *Vibrio* spp. infections that induce septicemia-related systemic inflammatory response symptoms [24, 43]. Skin necrosis is considered as the third-stage clinical presentation [9] and also as an important clinical feature of NF [8]. Skin necrosis can be found in 13.5% of all patients with NF and 27.9% of *Aeromonas* spp. NF cases [25]. Hemorrhagic bullae and necrotic cutaneous lesions were considered as the criteria for surgical intervention for NF [13], and they are also independent predictors of mortality of *Aeromonas* spp. NF cases [25]. NF caused by *Aeromonas* has been reported to have a high mortality rate (29.4%) in our 18-year retrospective study [25]. Although we found that *Vibrio* NF cases were more prone to develop hemorrhagic bullae and skin necrosis, we cannot ignore *Aeromonas* infection as these fulminant pathogens have the same clinical presentation and laboratory findings. About 14.6% of patients in the hemorrhagic bullae group presented with painless skin lesions (Table 3). Therefore, we must very careful to take the history about painless skin lesions besides bullae formation and cutaneous necrotic lesions at ED.

Furthermore, in our study, the hemorrhagic bullae group had more patients with band forms of leukocytes of more than 10%, anemia, thrombocytopenia, hyperlactatemia, higher serum creatinine level, and longer prothrombin time on arrival at the ED. This phenomenon may reflect the poor renal and hepatic dysfunction of patients with hemorrhagic bullae. The band forms of leukocytes of more than 10% were more common in the NF infected by Gram-negative than Gram-positive pathogens [24]. Hyperlactatemia reflected patients with hemorrhagic bullae combined with shock, respiratory failure, or renal failure [24].

In the past, we prescribed oxacillin and gentamicin as empiric antibiotics to treat suspicion of Non-Vibrio NF [31]. The initial selection of antibiotics for different infectious microorganisms that cause necrotizing fasciitis should be different. Based on the findings of the current study, for patients with no hemorrhagic bullae formation, we suggested ordering a third-generation cephalosporins combined glycopeptides for suspected no fulminate *Vibrio*, *Staphylococcus,* and *Streptococcus* NF. Third-generation cephalosporins combined with tetracycline which were commonly the empiric prescription before the infectious pathogens were identified when highly suspected fulminate *Vibrio* necrotizing fasciitis [10, 17].

Diagnosing NF is very difficult, because the cutaneous skin inflammation is found only at the ED, but in fact, the infection would have been rapidly progressed to the fascia layer. Delays in diagnosis and surgery of more than 24 h were found to be associated with increased mortality [2, 6, 33]. If we specially focus on bullae formation, it may warn us to be aware of this serious disease earlier. Early fasciotomy and early and appropriate antimicrobial regimen prescription should be performed for critically ill patients suffering from fulminant NF [6, 12, 40, 44] to save the patient’s life and limbs.

In conclusion, this study has suggested the following important points: (1) in southern Taiwan, patients with NF presenting with hemorrhagic bullae appeared to have the fulminant infective disease, more comorbidities and poor clinical outcome, including a higher amputation rate and more patients requiring postoperative intubation and ICU care than no hemorrhagic bullae groups, (2) patients with hemorrhagic bullae generally have a combination of bacteremia and *Vibrio* spp. infection, septic shock, and skin necrosis, (3) patients without hemorrhagic bullae generally occurred by culture-negative or gram-positive monomicrobial infection, and (4) if the physicians at ED can detect for the early symptoms/signs of NF as soon as possible, and more patient’s life and limbs may be saved.

## Limitations

This study was limited by having only 187 patients during a period of over 3 years and 5 months. Another limitation was that we assessed only the initial bleb types at the ED and further bullae conditions were not analyzed. The third limitation was that we compared only limb infection.

## Data Availability

The datasets analyzed during the current study are not publicly available, due to confidentiality reasons, but are available from the corresponding author on reasonable request.
